# Methods of ex vivo analysis of tissue status in vascularized composite allografts

**DOI:** 10.1186/s12967-023-04379-x

**Published:** 2023-09-08

**Authors:** Carolyn Ton, Sara Salehi, Sara Abasi, John R. Aggas, Renee Liu, Gerald Brandacher, Anthony Guiseppi-Elie, Warren L. Grayson

**Affiliations:** 1 Department of Biomedical Engineering, Johns Hopkins University, 400 North Broadway, Smith Building 5023, Baltimore, MD 21231 USA; 2 Translational Tissue Engineering Center, Johns Hopkins University, 400 North Broadway, Smith Building 5023, Baltimore, MD 21231 USA; 3 Department of Biomedical Engineering, Center for Bioelectronics, Biosensors and Biochips (C3B®), Texas A&M University, Emerging Technologies Building 3120, 101 Bizzell St, College Station, TX 77843 USA; 4 Department of Electrical and Computer Engineering, Center for Bioelectronics, Biosensors and Biochips (C3B®), Texas A&M University, Emerging Technologies Building 3120, 101 Bizzell St, College Station, TX 77843 USA; 5 Media and Metabolism, Wildtype, Inc., 2325 3rd St., San Francisco, CA 94107 USA; 6 Test Development, Roche Diagnostics, 9115 Hague Road, Indianapolis, IN 46256 USA; 7 Department of Plastic and Reconstructive Surgery, Vascularized Composite Allotransplantation (VCA) Laboratory, Reconstructive Transplantation Program, Center for Advanced Physiologic Modeling (CAPM), Johns Hopkins University, Ross Research Building/Suite 749D, 720 Rutland Avenue, Baltimore, MD 21205 USA; 8 Department of Cardiovascular Sciences, Houston Methodist Institute for Academic Medicine and Houston Methodist Research Institute, 6670 Bertner Ave., Houston, TX USA; 9 ABTECH Scientific, Inc., Biotechnology Research Park, 800 East Leigh Street, Richmond, VA USA; 10 Department of Chemical and Biomolecular Engineering, Johns Hopkins University, Baltimore, MD USA; 11 Department of Materials Science and Engineering, Johns Hopkins University, Baltimore, MD USA; 12 Institute for Nanobiotechnology, Johns Hopkins University, Baltimore, MD USA

**Keywords:** Vascularized composite tissue allografts, VCA, Biomarkers, Transplantation, Bioanalytical methods

## Abstract

Vascularized composite allotransplantation can improve quality of life and restore functionality. However, the complex tissue composition of vascularized composite allografts (VCAs) presents unique clinical challenges that increase the likelihood of transplant rejection. Under prolonged static cold storage, highly damage-susceptible tissues such as muscle and nerve undergo irreversible degradation that may render allografts non-functional. Skin-containing VCA elicits an immunogenic response that increases the risk of recipient allograft rejection. The development of quantitative metrics to evaluate VCAs prior to and following transplantation are key to mitigating allograft rejection. Correspondingly, a broad range of bioanalytical methods have emerged to assess the progression of VCA rejection and characterize transplantation outcomes. To consolidate the current range of relevant technologies and expand on potential for development, methods to evaluate ex vivo VCA status are herein reviewed and comparatively assessed. The use of implantable physiological status monitoring biochips, non-invasive bioimpedance monitoring to assess edema, and deep learning algorithms to fuse disparate inputs to stratify VCAs are identified.

## Introduction

Vascularized composite allotransplantation involves the transfer of anatomical structures containing multiple tissue types including skin, bone, fat, muscle, and connective tissue from one individual to another. Vascularized composite allografts (VCAs) range from face and hand to less commonly transplanted grafts such as the abdominal wall, uterus, and penis [1]. To date, more than 120 upper extremity and 46 face transplants have been performed worldwide along with other types of VCAs [2, 3]. Successful short- and long-term transplantation outcomes demonstrate VCA as viable treatment option for patients suffering large tissue defects or loss of limbs and for whom there are no conventional reconstructive options [4–7]. Currently, around 185,000 amputations take place in the US every year due to trauma, oncological resection, or severe burn, with the total number of amputees expected to reach 3.5 million by 2050 [8]. However, high immunogenicity and antigenicity are significant challenges in VCA, particularly with recipient response to skin. Acute rejection rates are approximately six-fold greater in VCA than in solid organ transplantation, necessitating treatment with immunosuppression and, in some instances, result in graft loss [1]. For amputees, limb prosthetics serve as an alternative to VCA transplantation. While prosthetics avoid the immunological challenges of graft rejection, functional and sensory recovery are unparalleled in VCA transplantation. Furthermore, prosthetics contribute to a significant rate of rejection as they are largely limited in practical applications, difficult to maintain, and awkward or uncomfortable to use [9]. While generally non-lifesaving, vascularized composite allotransplantation significantly improves an amputee’s quality of life, enables full functional recovery, and substantially recovers potential economic productivity loss [3, 10, 11].

Orchestrating the logistics of preserving and transporting the tissue remains a significant barrier to VCA accessibility. Once the graft is removed from circulation, a pathophysiological signaling cascade initiates due to ischemia and storage on ice. Under cold ischemia, grafts develop inflammatory signaling, muscle necrosis, mitochondrial dysfunction, and degraded vascular integrity [12–14]. Herzberg et al. and Piza-Katzer demonstrated that the extent of ischemic injury is inversely correlated with graft function in a bilateral hand transplantation study [15, 16]. Using the current gold standard method of static cold storage, the window of time for VCA viability is approximately 4–6 h [17]. This restricted time frame limits the geographic proximity of VCAs available for transplantation [18]. Compounding this constraint, strict aesthetic and anatomical features are necessary. While human leukocyte antigen (HLA) status is a metric for both organ and VCA suitability, anatomical criteria such as bone size, skin color, and soft tissue features are considered in VCA [19]. Prolonging the viability of VCAs beyond current constraints would therefore expand the availability of compatible allografts.

## Advancing machine-perfusion-based preservation

The gold standard in organ preservation, static cold storage, involves inducing hypothermia to reduce the metabolic demand of an organ or graft. Upon procurement, the VCA is flushed with a cold preservation solution and maintained on ice during transport. During this time, oxygen deprivation and the switch to anaerobic metabolism contribute to ischemic tissue injury [20]. In lowering the preservation temperature, static cold storage preserves the graft by slowing cellular metabolic activity [21, 22]. Despite its simplicity and widespread adoption in the clinic, static cold storage beyond a few hours is associated with early graft dysfunction in VCA due to tissue injury [20–22]. Muscle and nerve are particularly relevant to VCA preservation as these tissues are highly susceptible to ischemic damage [22–25]. In addition, once circulation is re-established and oxygen re-introduced to the now ischemic tissue, damage is exacerbated by reperfusion injury, a complex, injurious, pathophysiological cascade [13, 23, 26]. A number of detailed reviews on the molecular and cellular underpinnings of reperfusion injury have been published [12, 25, 27, 28]. Briefly, upon re-establishing blood flow, oxidative stress forms reactive oxygen species (ROS). Once the graft is transplanted, ROS and inflammatory signaling aggravate the innate immune system of the VCA recipient, leading to a cascade of tissue damaging events that encompass acute rejection. Furthermore, a study by Friedman and colleagues emphasized the contribution of injury-induced inflammation in acute allograft rejection [29]. Recently, machine perfusion has emerged as an alternative to ischemia that mitigates the consequences of metabolite accumulation and anoxia.

### Ex vivo perfusion

**Fig. 1:**
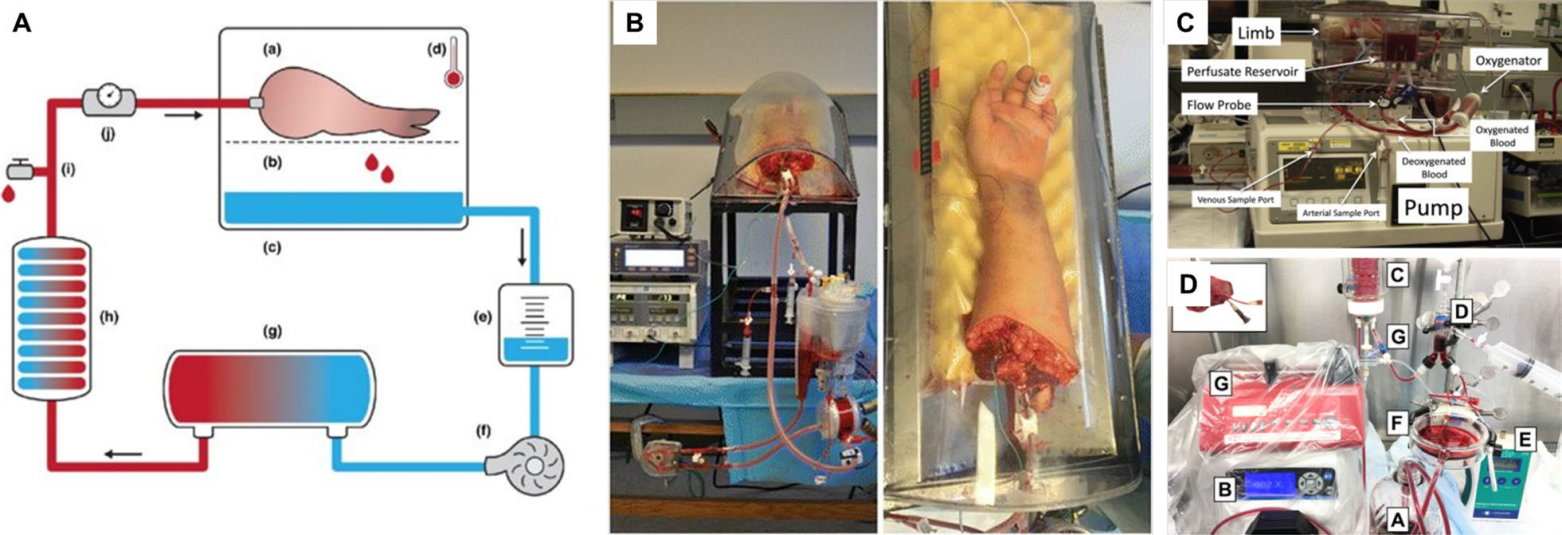
Perfusion bioreactor design and implementation. The general layout of the machine extracorporeal perfusion system commonly used in VCA ex-vivo perfusion. **A** A schematic illustration of the perfusate circulation as a system comprising **a** the porcine forelimb in the perfusion box on top of a **b** metal grid that allows **c** passive venous drainage of the preservation fluid, **d** a 15 mm needle probe that measures muscle temperature, **e** collection reservoir, **f** a centrifugal pump, regulating in-line pressure at ≤ 30 mmHg, **g** a membrane oxygenator, infusing the fluid with a mix of 95% O_2_ and 5% CO_2_, **h** heater-cooler machine, cooling the fluid to 8–10 °C, **i** drug administration point/fluid sampling port, and **j** flowmeter. (Image reproduced with permission from [54] ©2020 Anne Sophie Kruit et al. Transplant International published by John Wiley & Sons Ltd on behalf of Steunstichting ESOT.) **B** An actual perfusion system showing a human arm in the bioreactor chamber. **C** and **D** Photos of actual perfusion bioreactor systems consisting of an allograft housing, perfusate pump, perfusate oxygenator, heater, sampling port, and flow meter. (Images reproduced with permission from [47] © 2017 Wolters Kluwer Health)

Over the last few decades, ex vivo machine perfusion has presented promising options for prolonging organ preservation time. Figure 1 illustrates the design and implementation of a generalized machine perfusion bioreactor used in VCA. The intent of perfusion is to reduce the extent of ischemic damage by removing harmful metabolites while delivering nutrients to sustain cellular metabolism [30]. As a well-established focus in transplant medicine, detailed reviews of organ preservation solutions and their compositions have been published [31, 32]. Generally, perfusates include the following components: colloids to minimize edema by increasing oncotic pressure, ions to preserve membrane function or serve as buffering agents, saccharides to sustain glycolysis and/or increase osmotic pressure to counteract edema, and dilute red blood cells or artificial hemoglobin to facilitate oxygen transport [30, 31, 33]. Furthermore, sodium bicarbonate is included to balance metabolite accumulation and insulin is included to increase glucose uptake [17, 20, 34]. Perfusion typically occurs through attachment to intact arteries and vessels to pulsatile perfusion systems following [18]. In early studies, pumping perfusate through grafts led to significant pressure-induced tissue injury, or barotrauma [35]. Nonetheless, there has been noteworthy progress and even commercialization of ex vivo perfusion systems designed for solid organs including the kidney, liver, lung, and heart [36]. Much research over the last 30 + years has focused on adapting solid organ perfusion systems for VCA preservation [37–41]. In experimental VCA, perfusates adapted from solid organ transplantation such as STEEN Solution™, Perfadex®, Custodiol® HTK, Celsior®, and University of Wisconsin (UW) Solution have been used to perfuse tissue with comparable efficacies [42–45]. Blood-based perfusates have also been used to facilitate oxygen delivery [41, 46]. Promising outcomes from large animal models encouraged Werner and colleagues to study ex vivo perfusion in human VCA, subsequently demonstrating the feasibility of human limb machine perfusion for 24 h with plasma-based hemoglobin [47]. In a surgical setting, VCA is typically perfused prior to static cold storage with UW solution established as the preferred perfusate. However, the empirical rationale for its selection has not been thoroughly characterized [30, 48]. While differences in transplant outcomes have been compared in solid organ preservation, comparative data in VCA is limited and there currently is no consensus on optimal perfusate composition [49–53]. Despite advances in VCA perfusion methodology, corresponding metrics to evaluate their efficacy need to be identified in order to catalyze advancement of the field.

## Surrogate biomarkers of VCA injury during preservation

Within the complex tissue architecture of VCA, nearly imperceptible damage occurs during preservation. Vascular degeneration, necrosis, and metabolite accumulation include those changes which are not visually detectable yet lower the likelihood of successful transplant outcomes. Evaluation of tissue integrity and pathology is typically achieved with tissue biopsy and subsequent histology. Using this method, features such as cellular muscle structure and edema can be visualized with high resolution. The Banff Criteria ranks tissue pathology in VCA and encompasses a set of histopathological features that distinguish severity by the extent and localization of immune infiltrate (Table 1) [55, 56]. Enhancing its resolution, Rosales and colleagues have expanded on the Banff Criteria to stratify epidermal and vascular tissue features [57]. Immunostimulatory molecules evoked by surgical tissue damage and altered metabolic activity under ischemia trigger the infiltration of macrophages into muscle and skin [56, 58]. While the resolution of tissue histology may yield invaluable insight for VCA status, applications to ex vivo graft preservation are limited due to its invasiveness, time-intensiveness, intrinsic inter- and intra-observer variability, and lack of quantification. In one center, it was found that approximately 80% of in vivo skin containing VCA rejection episodes that were resolved with topical therapy or oral immunosuppression were scored as Grade III using the Banff Criteria [59]. Interestingly, VCA with rejection episodes necessitating systemic administration of immunosuppression were also scored as Grade III. To overcome the uncertainty associated with histopathological grading, quantifiable VCA features and alternative biomarkers of viability have emerged in recent time. A review of the literature that employs a range of tissue monitoring methods was conducted using Scopus and PubMed. Given the relatively recent development of VCA surgery, all original research articles up to the present were included. Search terms included ‘vascularized composite tissue allograft/allotransplantation’ in combination with the following terms: biomarker, monitoring, diagnostics, perfusion, viability, rejection, ex vivo preservation.

**Table 1:** Banff classification of acute rejection in skin-containing allografts

Grade 0		No or rare inflammatory infiltrates
Grade I	Mild	Mild perivascular infiltration. No involvement of the overlying epidermis
Grade II	Moderate	Moderate-to-severe perivascular inflammation with or without mild epidermal and/or adnexal involvement (limited to spongiosis and exocytosis. No epidermal dyskeratosis or apoptosis
Grade III	Severe	Dense inflammation and epidermal involvement with epithelial apoptosis, dyskeratosis, and/or keratinolysis
Grade IV	Necrotizing acute rejection	Frank necrosis of epidermal or other skin structures

**Fig. 2:**
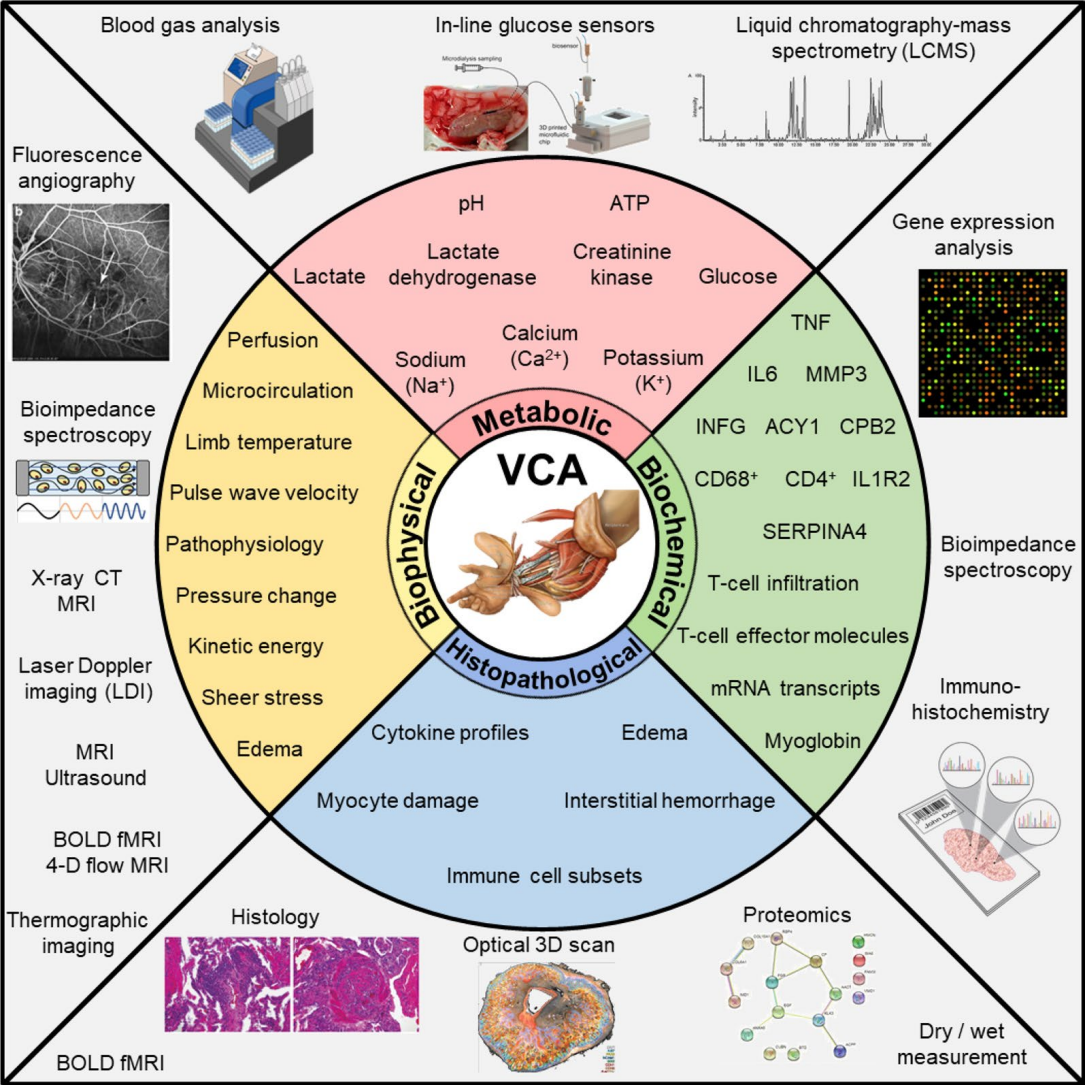
An illustrative summary of methods of analysis of tissue status during preservation of allografts in VCA. Clockwise: Metabolic, Biochemical, Histopathological, and Biophysical. (Images reproduced with permission from [60, 61] © 2015 Royal College of Ophthalmologists, 2019 American Chemical Society, respectively.)

Figure 2 provides an illustration that summarizes the various methods of analysis of tissue status during preservation of allografts in VCA. The illustration follows the methods described in the following sections.

### Metabolic and biochemical biomarkers

During solid organ perfusion, cellular metabolites are often used as surrogates of transplant viability. With effective machine perfusion, perfusate carries biomolecules from the interstitial space for sample collection and analysis. These surrogate viability markers are derived from normal metabolic processes, whose deviation from established physiological values are thought to indicate graft deterioration. Once the VCA is removed from the donor, the surgical tissue damage, the ischemic period and lack of circulation alters the graft on a molecular and cellular level. Oxygen and nutrient deprivation lead to series of ionic and metabolic changes in cells over time. Membrane potential and ion compartmentalization can be maintained with low ATP reserves early in ischemia, however, after a few hours, mitochondrial activity is altered and membrane potential decreases [14]. Without blood flow, cells are still able to synthesize ATP through anerobic metabolism and phosphocreatine (PCr) pathways, such that ATP drops slowly as PCr and glycogen remain abundant. However, three hours into ischemia, ATP declines quickly, followed by complete consumption of PCr and glycogen 6 h post ischemia [62]. The switch from aerobic to anaerobic metabolism has implications in metabolite accumulation that may give insight to graft viability and functionality. Upon ischemia, the change in cellular metabolism result in the accumulation of NAD, lactate, and H^+^, thus lowering intra- and extracellular pH [23]. Furthermore, lactate, creatine kinase, potassium, lactate dehydrogenase have been identified as indicators of ischemia [63]. Lactate concentration is commonly used as a surrogate marker of cellular stress and hypoxia in solid organ transplantation and is often used as a marker for VCA viability [20, 43, 64, 65]. Lactate above a concentration of 5 mmol/L at the end of the heart perfusion has been suggested as an indicator of poor post-transplant outcomes [66]. In a human limb tourniquet study, lactate reportedly increased to 28 mmol/L after 36 min of ischemia, subsequently 15 mmol/L has been suggested as initial estimate for VCA lactate threshold [67]. Acidosis is associated with reduced VCA functionality for ex vivo timepoints, specifically in studies which use muscle contractility as another metric of viability [68]. Additional study of the correlation between pH and VCA outcome is needed to bolster biomarker efficacy.

Concurrently, glucose, phosphocreatine, and ATP content of tissue inform its rate of energy consumption. As graft viability deteriorates, metabolism is expected to slow to an eventual stop. Liquid chromatography-mass spectrometry (LCMS) as well as blood-gas analysis have been used to quantify metabolites contained in tissues or perfusate following static cold storage or perfusion. Compared to a warm treatment group in a rat hindlimb model, Gok and colleagues demonstrated that static cold storage incurred the greatest decrease in phosphocreatine and creatine using LCMS [22, 69]. However, a significant difference in ATP content or the ratio of ATP/ADP was not evidenced in either study conducted. In another study, similar findings indicated a non-statistically significant increase in glucose content of cold stored VCA after 6 h of perfusion [20]. More recently, in-line glucose sensors have been applied to organ perfusion to continuously monitor perfusate [70]. Despite some encouraging evidence of glucose as a metric of cellular metabolism, it may be challenging to use as a biomarker when added to perfusate. This issue necessitates the use of more sensitive glucose sensors as well as precise control of perfusate composition.

Calcium is a key mediator of muscle contraction. During tissue and organ preservation, ischemia induces mitochondrial dysfunction and alters cellular calcium retention [71–75]. This process has been characterized in cardiac transplantation and is associated with impaired organ function [71, 76]. In VCA, metabolically-active skeletal muscle is highly susceptible to ischemic injury. Depletion of ATP inhibits ion exchange by sodium–potassium ATPases as well as calcium ATPases [72, 75]. Consequently, sodium-calcium antiporters reverse in mechanism to restore cytosolic sodium concentration, leading to an accumulation of cytosolic calcium [72]. Sodium, calcium, and potassium concentration are often used as a measure of ischemia in VCA. Specifically, increased intracellular ion retention has been used to identify the onset of tissue ischemia during preservation. In a study by Amin and colleagues, porcine forelimbs were preserved under an extended period of static cold storage or perfusion with varying temperatures and mean arterial pressures for a total of 8 h prior to transplantation and reperfusion [20]. During reperfusion, blood-gas analysis revealed a destabilized electrolyte profile and acidosis in the static cold storage group, this was treated with repeat infusions of bicarbonate to prevent graft loss. These limbs demonstrated depleted sodium and calcium levels and increased potassium relative to the machine perfusion groups.

The consequences of tissue damage due to ionic metabolite imbalance are not well-understood. Despite an apparent connection between VCA viability and metabolic activity, measurement of biochemical parameters is confounded by the lack of standardized methods in preservation. Differences in graft model, ischemic duration, perfusate composition, and perfusion parameters such as flow rate and pressure may influence the accumulation of metabolites. In machine perfusion systems, metabolite accumulation in the perfusate may indicate the status of cellular metabolism and severity of ischemic injury. Perfusate choice may also affect metabolite measurement yet has not been comparatively assessed in VCA. In one study, the impact of perfusate composition was shown in relation to lactate abundance. Kruit and colleagues demonstrated lactate level in abdominal wall machine perfusion perfused by UW was higher than tissue perfused with Custodiol®HTK, which may empirically reflect the intended formulation of UW to support cellular metabolism, whereas HTK is designed to stabilize membrane potential and minimize cell activity [77]. In order to use cellular metabolites as surrogates of tissue injury, further research should consider contributions of perfusate composition and biophysical parameters to quantified tissue status. The forgoing suggests that continued development of fully intra-muscularly indwelling physiological status monitoring biochips that measure metabolites such as glucose, lactate, pH (acidosis) and potassium promises to be welcomed additions to the VCA metabolite monitoring arsenal [78–84].

### Biophysical biomarkers

Once grafts are removed from the donor and subject to static cold storage or perfusion, the biophysical properties of composite tissues become substantially altered. In organ transplantation, donor history including age, illness, medication regimen, and cause of death have been used to confirm acceptability. Studies applying machine perfusion to organs from non-heart-beating donors (NHBD) indicate perfusion pressure and vascular resistance as markers of graft status and perfusion efficiency. In a study of NHBD kidneys, pulsatile hypothermic perfusion precedes a reduction in renal vascular resistance, improved cortical flow, and clot ejection from microcirculation [20, 22, 68, 85, 86]. These observations support that machine perfusion enabled effective perfusate delivery. In a similar study, ischemic kidneys had an elevated vascular resistance (1.25 mmHg/mL/min) compared to a perfused group (0.75 mmHg/mL/min) [87]. Furthermore, warm ischemia time has been shown to increase the risk of delayed graft function in NHBD organs, which may be due to acute tubular necrosis [85]. Vascular resistance in NHBD kidneys has also been investigated as prognostic factor of transplantation outcome in combination with donor history parameters. Kidneys transplanted with low vascular resistance after 20–30 h of hypothermic perfusion with UW have shown improvement in graft survival comparing to average NHBD kidneys survival [88, 89]. Intra-organ vascular resistance at the beginning of perfusion has been demonstrated to directly correlate with warm ischemia time, which may be mitigated by prolonging machine perfusion prior to transplantation [90].

Under native physiological conditions, the endothelium maintains vascular homeostasis. The elasticity of intact blood vessels and capillaries facilitates an adaptive response to varying hemodynamic pressures [91]. As a physiological analog to electrical circuits, vascular resistance is the opposition to hemodynamic flow [92]. Generally, organ machine perfusion systems follow pressure-controlled protocols. The lower end of physiological blood pressure has been shown as optimal to prevent tissue edema [17, 93]. This parameter is influenced by vessel dimensions, perfusate viscosity, flow rate, and temperature [20, 41, 50, 91]. Additionally, perfusate colloid concentration has been shown to affect vascular resistance. In kidneys perfused with a varying colloid concentrations, vascular resistance was observed to be higher in kidney perfused with lower colloid perfusate in comparison with perfusate containing higher colloid [94]. Change in vascular resistance is often used as a surrogate for perfusion efficacy and vascular integrity. Whereas decrease in resistance may occur due to barotrauma and vascular collapse, stable resistance may indicate intact vasculature. During extended limb perfusion with a pressure-controlled circuit, vascular resistance tends to decrease within an hour of perfusion and stabilize thereafter [34, 43, 47]. Gok and colleagues demonstrated that a perfusion flow rate of approximately 1 mL/min lead to a gradual increase in perfusion pressure, barotrauma, lactate, and limb edema [43]. In another instance, a perfusion circuit by Fahradyan and colleagues increased flow rate until a physiological arterial pressure of 102.9 ± 1.76 mmHg was reached [68]. At the end of perfusion, vascular resistance was found to be increased by 6.42% ± 18.41%. The authors posit that the observed increase in vascular resistance was due to vascular spasm, endothelial edema, and microvascular collapse leading to perfusion failure [68]. Generally, regardless of temperature, perfusate and organ intra-organ vascular resistance during long-term perfusion has been recorded as high at the beginning of machine perfusion but declines and stabilizes as perfusion continues beyond 1 h. Due to differences in experimental conditions and perfusate composition, a significant change in any of these parameters during perfusion may impact pressure reading [95]. Global tissue physiology and comparative analysis of perfusate parameters must be considered in the evaluation of vascular resistance as a surrogate for tissue status.

Aside from ensuring tissues are viable, they must also be functional for successful VCA transplantation outcomes. Due to the accumulation or imbalance of key ions and metabolites, muscle tissue contractility and graft function deteriorate [47, 69, 86]. Namely, allografts such as the face and limbs must enable muscular capabilities in the recipient. Ex vivo VCA tissue assessments attempt to validate functionality by testing single-muscle contractility, where a decay in force generated by the muscle fiber is a sign of reduced graft function. This measurement is often taken by excising and permeabilizing single muscle fibers, then placing them in a calcium-containing solution. Single fibers are dissected then secured to a force transducer. Some researchers have assessed contractional force by immersing tissue in solutions containing high concentrations of calcium and ATP [47, 86]. Alternatively, muscle contraction has also been induced by using point electrodes to deliver a supramaximal electrical stimulus to whole ex vivo limbs [22, 69]. The force generated is normalized to the cross-sectional area of the muscle and used to estimate fiber contractility and functionality. Constantinescu and colleagues found that perfused porcine forelimbs maintained a motor response to electrical stimulation for the duration of 12-h perfusion, whereas non-perfused tissue was unresponsive after 30 min [41]. Across experiments comparing muscle contractility in static cold stored and perfused grafts, the latter has been shown to sustain muscle contraction for up to a maximum of 12 h [69, 86]. Furthermore, it was found that reattachment of static cold stored limbs did not recover force generation, indicating that perfusion may be better suited for long-term ex vivo VCA preservation [86]. A correlation between the duration of cold ischemia on limb function has been investigated by Tsuji and colleagues, who histologically identified muscle degeneration and necrosis in rat hind limbs maintained at 4 °C for up to 72 h [96]. The findings were validated using electromyography 3 weeks following transplantation, which indicated a longer delay in motor response for all ischemic groups compared to limbs transplanted immediately. In a separate functional validation, one group used porcine abdominal muscle flaps with electrical field stimulation used for recording muscle contraction [97]. This inherently non-contact electrical field stimulation approach may be better suited for graft transport during ex vivo preservation [41, 97]. As a whole, muscle contractility is surrogate for graft function that may inform long-term transplant success, although factors such as preservation condition, ischemic and/or reperfusion damage, and the integrity of nerves and muscles must be considered with tissue force generation to identify causes of reduced tissue functionality. The continued development of multiplexed multielectrode bioimpedance spectroscopy used in the measurement of edema, with and without electric field stimulation, may be a welcomed addition for the continuous monitoring and stratification of VCA status during preservation [98–101].

### Histopathology and tissue composition biomarkers

**Fig. 3:**
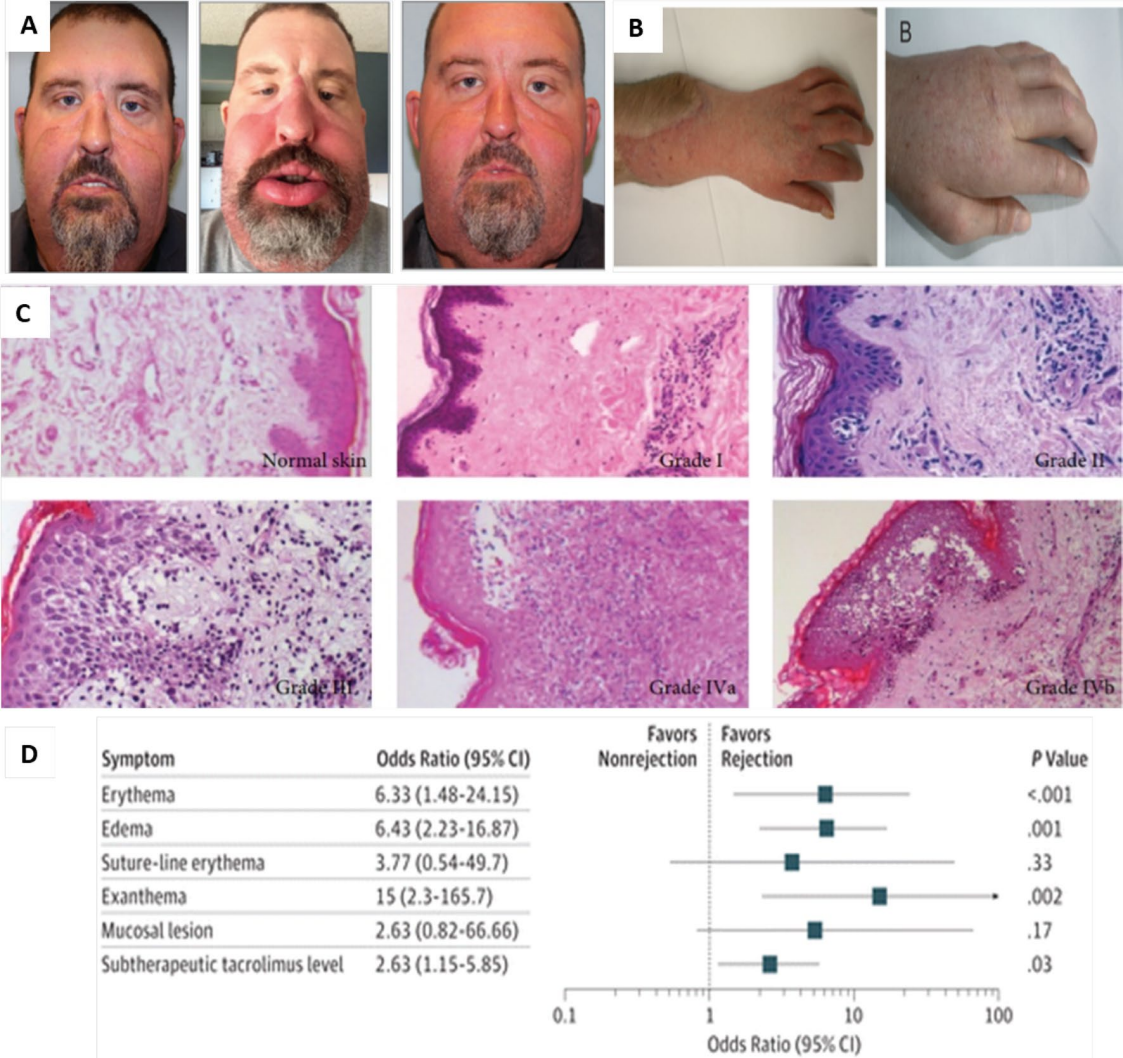
Edema is the main Indicator of acute rejection in VCA. **A** Representative images of edema manifestation in face allograft acute rejection i. No rejection (POM21), ii. early rejection (POM 8) and iii. late rejection (POM26). (Images reproduced with permission from [2] © 2019 Mary Ann Liebert, Inc.) **B** Edema manifestation in hand allograft acute rejection (Images reproduced with permission from [107] © John Wiley & Sons, Inc.) **C** Skin allograft acute rejection graded based on Banff Scoring System. Normal skin: unaffected skin, GradeI: mild perivascular infiltration, GradeII: mild perivascular infiltration with/without mild epidermal or adnexal involvement. No epidermal dyskeratosis or apoptosis, Grade III: dense inflammation and epidermal involvement with apoptosis, dyskeratosis, and/or keratinolysis, Grade IV: necrotizing acute rejection. necrosis of skin structures. (Images reproduced with permission from [108] © 2013 Ravi Starzl et al.) **D** Association of clinical signs or subtherapeutic tacrolimus levels with acute rejection episodes. (Image reproduced with permission from [2] © 2019 Mary Ann Liebert)

Aside from biochemical and biophysical VCA components, additional factors within the tissue may inform graft viability. Markers of inflammation such as edema, cytokine profiles, and immune cell subsets have been explored as potential biomarkers in VCA. In skin-containing allograft preservation and transplantation, edema is commonly used as an indication of graft injury or rejection [55, 57, 102–104]. Typically, during experimental VCA, samples of tissue are isolated then desiccated, the initial “wet” weight and subsequent “dry” weight yield a ratio used as a surrogate metric of edema [105]. Alternatively, histology is commonly used to increase the resolution of tissue assessment and identify edema on a cellular level. Figure 3 is a collage illustrating the significance of edema as a clinical indicator of acute rejection in VCA. In a porcine skin-containing VCA flap model, Rosales and colleagues used histology to determine extent of inflammation, as evidenced by immune infiltrate and edema [57]. From transplant recipients of either haploidentical or class I MHC mismatched groups, 28 serial biopsies were taken from 8 animals and ranked by the relative amount of inflammatory infiltrate for correlation to graft survival time. Biopsies from the mismatched transplant indicated a greater presence of immune cells as well as marked edema compared to the matched group where mild edema was noted. The MHC mismatched transplants were ultimately rejected. While the scoring methodology delineated relative degrees of inflammatory infiltrate, the correlation of edema with graft survival lacks quantitative rigor. Therefore, the extent of edema across the two groups was difficult to associate with graft survival. In a swine hindlimb transplantation model, Etra and colleagues applied the Banff grading system to characterize skin rejection [55]. Grafts that exhibited a pattern of severe edema, erythema, and local inflammation were stratified for abandonment rather than treated with immunosuppressive therapy. This was due to the purported low feasibility of graft rescue. Furthermore, the authors noted that the Banff grading criteria was not sufficient for categorizing the range of graft conditions observed. Additionally, some edematous transplants recovered from mild tissue swelling, thus indicating that more quantitatively rigorous VCA stratification may be necessary [106]. Quantifying the extent of edema may identify a definitive correlation between tissue swelling and graft outcomes, thereby expanding the toolkit of biomarkers applicable to ex vivo VCA [99, 100]

In solid organ transplantation, in vivo biomarkers have been explored as surrogates to predict long-term survival [109–112]. Components present in blood or serum are often targets for indirect biomarker discovery due to the inherent challenge of sampling from internal organs. For example, blood accumulation of myoglobin, a protein that binds oxygen to facilitate its diffusion in muscle, has been used as a surrogate for cardiac and skeletal muscle injury [113, 114]. In VCA, limited studies have explored non-invasive biomarkers for tissue viability [115]. Honeyman and colleagues have described the following biomarkers identified in VCA: T cell infiltration, T cell effector molecules, and mRNA transcripts of genes associated with rejection [115]. Similarly, Puscz and colleagues have assessed histological and immunological indicators in their study of rat hindlimb transplantation [116]. Chronic rejection (CR) in grafts was identified as the appearance of erythema or edema and subsequently rescued with cyclosporine A and dexamethasone. Compared to non-immunosuppressed rats and those receiving constant cyclosporine A, the CR group exhibited intimal hyperplasia with an approximate three-fold increase in proliferation of intimal cells within the femoral artery. Additionally, immunohistochemistry (IHC) revealed an increased accumulation of CD4^+^ and CD68^+^ cells in the CR group. Gene expression analysis of interleukin 6 (IL6), tumor necrosis factor (TNF), and interferon gamma (IFNG) indicated a statistically significant increase in IFNG. In cytokine microarrays, upregulation of various interferon genes was observed in the CR group. In a similar manner, proteomics in chronic rejection has been explored in vivo. Kollar and colleagues evaluated longitudinal serum samples from face transplant recipients to identify potential proteins of interest [117]. The following were found to be highly abundant in instances of severe rejection: MMP3 (Matrix Metalloproteinase 3), ACY1 (Aminoacylase-1), IL1R2 (Interleukin-1 receptor type 2), SERPINA4 (Kallistatin) and CPB2 (Carboxypeptidase B2). As an advantage to IHC or biopsy, proteomic analysis enables non-invasive detection of potential markers of tissue status. The novelty of using proteomic discovery tools in VCA biomarker research presents a promising non-invasive method to evaluate tissue status in vivo, and potentially during ex vivo preservation.

Circulating donor-derived DNA and microRNA is another direction for biomarker development that has been explored in solid organ transplantation and in vivo VCA [118–121]. When cells and tissues of the donor allograft degrade in vivo, fragmented nucleic acids release into the bloodstream and are cleared within 30 min to two hours. The resulting cell-free donor DNA can be detected in blood or urine [121–123]. In a preliminary study, Haug and colleagues quantified cell-free donor-derived DNA from plasma samples from a face transplant recipient as well as a bilateral arm transplant recipient [124]. The accumulation of cell-free donor-derived DNA was correlated with rejection, although its increased concentration was not solely dependent on rejection onset. While measuring cell-free DNA is non-invasive and quantitative, detection sensitivity and enhanced understanding of cell degradation in transplanted VCA is still under refinement. In infectious disease and cancer, microRNAs (miRNAs) have long been a source of interest as potential diagnostics [125–128]. In particular, circulating nucleic acids such as miRNAs can serve as evidence for pathology due to its highly specific and unique signature [129]. Recently, Di Stefano and colleagues have reviewed miRNAs identified in VCA for the detection of allograft rejection [130]. Various miRNAs with immunomodulatory or effector roles were described with their relation to incidence of rejection. In one study, graft deterioration due to ischemia was discussed in the context of myocardial injury, however a correlation was not observed for the prognostic miRNAs identified. Despite significant developments in miRNA applications to solid organ transplantation, comparable progress in VCA is lacking [131–133]. Nonetheless, the prognostic modality has potential for limited applications in ex vivo tissue assessment such as screening for immunogenicity and ischemic damage.

## Non-invasive VCA evaluation methods

Clinically relevant avenues to expand upon the applicability of VCA biomarkers include spatial capability, continuous feedback, and real-time quantitation. Medical imaging technology confers these properties and has been applied to ex vivo and in vivo VCA analysis. Conventional methods including x-ray, computed tomography (CT), magnetic resonance imaging (MRI), and high-resolution ultrasound have been leveraged to visualize tissues on a cellular level. An increased resolution in VCA monitoring has been used to reveal dynamic processes such as microcirculation and perfusion, relative change in cellular metabolism, and the development of pathophysiology. These methods have been used primarily for peri-operative monitoring of in vivo VCA [134]. Nonetheless, medical imaging presents advantages to existing VCA biomarkers and has the potential for widespread clinical utility, particularly for monitoring tissues during preservation (Table 2).

**Table 2:** A comparison of clinically relevant features of VCA assessment modalities

Technique	Non-destructive	Portable	Rapid	Continuous	High Res	Quantitative	Facile analysis	Low cost	Refs.
Histology	○	○	○	○	•	○	•	•	[55, 200]
Wet:dry weight	○	•	○	○	○	•	•	•	[105, 201]
Optical 3D scanning	•	•	•	○	○	•	•	•	[150, 202]
OCT	•	○	○	○	•	○	○	○	[145]
Laser Doppler imaging	•	•	•	•	○	◒	○	•	
Thermographic imaging	•	•	•	•	○	•	•	•	[138–140, 143]
NIR lymph-angiography	•	○	•	•	•	◒	•	○	[146, 149]
X-ray	◒	•	•	○	○	○	○	•	[144]
CT and µCT	◒	○	○	○	•	◒	○	○	[144, 155, 156, 181]
fMRI	•	○	○	○	•	◒	○	○	[160, 162, 163]
Ultrasound	•	•	•	○	•	◒	•	•	[170, 171, 173, 174]
Bioimpedance spectroscopy	•	•	•	•	◒	•	◒	•	[184, 195, 203]

• Demonstrated to be valid in experimental and/or clinical settings

○ Not applicable to the listed detection method

◒ Partially applicable

### Imaging in the infrared and visible spectrum

In VCA transplantation, vascular patency and occlusion are parameters relevant to graft failure as perfusion is associated with microcirculatory damage [135]. For this reason, methods to assess VCA tissue leverage flow dynamics to quantify microcirculation [136, 137]. Laser Doppler imaging (LDI) has been used to dynamically visualize vasculature, blood flow, and perfusion in superficial tissues [138, 139]. In a rat hind limb free flap transplant model, the method has been used to visualize tissue perfusion to diagnose vascular stenosis in VCA in vivo*.* [140]. Similarly, thermographic imaging has also been used for the noninvasive, non-contact detection of vascular disorder and inflammation in vivo*.* [68, 141, 142]. It has also been used to monitor limb temperature during perfusion to verify normothermia [68]. Recent advancements in hardware have simplified thermographic measurement to enable rapid data acquisition using smartphone-compatible technology [143]. While vascular integrity and neo-angiogenesis are relevant markers in vivo, pre-transplantation biomarkers of VCA remain unestablished. In order to elucidate fluid dynamics of VCA during preservation, near-infrared detection methods have been used to develop models of pathology [139]. Building upon optical technology, near-infrared (NIR) methods including ICG lymphoscintigraphy and lymphangiography enable deeper light penetrance into tissue [139, 144]. For example, ICG lymphangiography can be used to track real-time fluid movement in VCA for the quantification of lymphatic drainage [145, 146]. Lymphangiography involves administration of contrast agents and requires high technical skill to accomplish delivery via cannulation. Similarly, fluorescence angiography with indocyanine green (ICG) has been used to visualize microcirculatory and peripheral perfusion in VCA [68, 147]. Although fluorescence imaging has high diagnostic sensitivity, signal attenuation in deeper tissues may occur due to limited light penetrance and scattering [139, 148, 149]. Furthermore, ICG can bind proteins present in the fluid, leading to reduced image resolution [146]. Nonetheless, this method non-destructively enables real-time visualization of fluid circulation in ex vivo models of VCA, which may be used to indicate perfusion efficacy.

Imaging methods that use visible light are semi-quantitative and have relatively minimal hardware requirements. Optical 3D scanning using camera-based measurement has been applied experimentally for the quantification of leg edema in vivo [150]. Parameters including leg curvature and instep height can be derived from 3D coordinates in order to correlate measurements to fluid retention. However, confounding factors such as patient activity and measurement error based on selected reference points reduce reliability (r = 0.64). Optical coherence tomography (OCT) increases the imaging resolution to a micrometer scale and is commonly used to detect macular edema [151–153]. In soft tissue, OCT has been applied to temporally quantify edema in a mouse ear burn model [145]. However, due to the use of visible light, this method has low penetrance of non-opaque tissues [145]. While OCT has been demonstrated in macular and auricular tissues to detect interstitial fluid accumulation, this method currently has not been demonstrated for use in VCA.

### X-ray and computed tomography

Under appropriate contrast enhancement, X-ray and computed tomography (CT) enable visualization of soft tissue features such as blood vessels, blood clots, and tumors [154]. MicroCT (µCT) is an application of X-ray imaging with miniaturized hardware and enhanced resolution. The technology is commonly used to visualize vasculature in high resolution and has been used in ex vivo VCA, namely to visualize tissue perfusion and pressure-induced damage and to inform perfusion regimens during preservation [155]. While µCT imaging can measure surrogates of transplantation success, such as neo-vascularization, its utility in ex vivo VCA has not been thoroughly established. Furthermore, long data acquisition times of multiple hours for dense tissue, limits its applicability in VCA preservation. Additionally, evaluating cellular and molecular tissue details requires the injection of molecular agents to increase soft tissue contrast [139, 156]. Despite its current limitations, the high resolution spatial information provided by µCT yields prognostic value in a preservation setting and the miniaturized technology is deployable for ex vivo monitoring.

### Magnetic resonance imaging

Magnetic resonance imaging (MRI) provides high spatial resolution and extensive visualization of soft tissue [157]. In T2-weighted images, edema can be detected in soft tissues using standardized procedures [158, 159]. Subsequently, T2-weighted methods can assess edema for VCA in vivo*.* [160, 161]. Beyond traditional MRI, functional MRI (fMRI) techniques reveal dynamic processes. This method has been used to obtain spatiotemporal information in kidney transplantation, myocardial infarction, and cerebral tumors for edema detection [160, 162, 163]. In VCA, the functional MRI method of flow MRI has been used for longitudinal follow-up of transplant vascularization by measuring intravascular flow [164]. In contrast to X-ray or CT methods, flow MRI can be used to evaluate hemodynamics in the absence of an injected contrast agent, thereby minimizing shortcomings associated with uptake of contrast agents by tissue. Alternatively, blood-oxygen-level-dependent (BOLD) fMRI has been presented as a novel, noninvasive alternative to oxygen-sensing electrodes for obtaining prognostic metrics in vivo*.* [165]. Oxygen saturation measured by BOLD fMRI may serve to illuminate VCA reperfusion-injury to elucidate mechanisms of graft deterioration. Expanding upon this method, four-dimensional flow MRI increases data acquisition to derive hemodynamic parameters such as shear stress, pressure change, turbulent kinetic energy, and pulse wave velocity [166]. This has been applied extensively in cardiology to obtain detailed images of blood flow through the cardiovascular system and may be adapted to assessment of VCA perfusion efficacy and vascular integrity [167]. However, the high resolution images obtained by fMRI necessitate powerful equipment. MRI machines of up to 7 Tesla were used in aforementioned hemodynamic studies. Currently, MRI miniaturization, while being aggressively pursued, has not reached the stage of portability needed for transplant monitoring [168]. Subsequently, MRI serves more effectively to assess VCA transplants in vivo for long-term study of vasculature.

### Ultrasound imaging

The most significant advantages of ultrasound over MRI are the widespread access to ultrasound machines and feasibility of real-time tissue assessment. In one instance, ultrasound biomicroscopy identified markers of chronic rejection in in vivo VCA by detecting transplant arteriopathy in face transplants [169]. The high resolution of ultrasound biomicroscopy enables measurement of blood vessel wall thickening, currently explored as an indicator of graft vasculopathy and potential marker of chronic rejection [170, 171]. Ultrasound has also been used to intraoperatively assess blood flow patency on the day of surgery and one week post-operatively to ensure blood vessels have been successfully connected [172]. Measurements of blood flow and vessel structure were used to demonstrate that the microsurgery and perfusion with blood did not induce subsequent graft deterioration. The high-resolution imaging afforded by ultrasound biomicroscopy is a promising application to allograft monitoring and has potential to be leveraged for ex vivo applications, although suitability for the complex tissue architecture of VCA has not been established.

A limited number of novel developments strive to leverage ultrasound to quantify observations of edema and to measure the depth of pitting edema [173–175]. In a clinical study, the relationship between surface imprint depth, circumferential measurement, and tissue thickness revealed that depth of surface imprints correlate with subcutaneous tissue thickness by a coefficient of 0.736 [174]. In a similar manner, Pitre and colleagues considered the viscoelasticity of tissue as a measure of edema status and simplified hardware by designing a single-element ultrasound transducer [175]. Graphing tissue viscoelasticity across simulated edema conditions indicated depth dependency, therefore providing enhanced quantification of superficial edema. Ultrasound-based prognostic methods provide advantages of being widely available, nondestructive, portable, and simple to use (Table 2). However, ultrasound analysis requires expert knowledge of anatomical features and requires further analysis for quantification. Furthermore, efforts to correlate echogenicity with edema have been limited to superficial tissues, emphasizing that sufficient resolution in deeper tissue has not been established. While moving toward clinically accessible technology, the feasibility of ultrasound in ex vivo transplant medicine is currently limited by the lack of robust quantification and hardware compaction yet has promise as a non-destructive and high-resolution tissue monitoring tool.

### Bioimpedance in monitoring and imaging

Bioimpedance spectroscopy (BIS) and electrical impedance tomography (EIT) are methods entrenched in diverse fields including agriculture, nutrition, and cellular biology [176–178]. To measure bioimpedance, an electric current is injected through the tissue then potential difference is collected [99]. Bioimpedance is calculated as the ratio of the injected current and collected potential. By varying the frequency of the current, specific cellular compartments can be probed. At lower frequencies, the current flows through the extracellular space. As frequency increases, current passes through the cell membrane and into the intracellular space [179]. Properties such as tissue density, cellular integrity, and fluid accumulation contribute to the total impedance. The application of bioimpedance for edema detection is a well-established method in lymphedema diagnosis [180]. Additionally, bioimpedance sensing has been used to monitor edema manifesting in cerebral hemorrhage, colitis, and in superficial tissue [148, 181–186].

The current range of noninvasive methods to evaluate VCA includes high-contrast, spatiotemporal imaging sufficient to resolve relevant pathophysiological tissue features such as vasculopathy and interstitial edema. However, these methods often require sophisticated instrumentation and specialized equipment. Bioimpedance analysis uses relatively minimal instrumentation for quantitative tissue assessment based on intrinsic electrical properties, with subcellular and tissue resolution depending on applied frequency ranges [187–193]. Limitations to bioimpedance applications in VCA include the lack of standardization in protocols, as well as unknown baselines for parameters measured. Standardization including optimal electrode placement, frequencies for measuring VCA impedance, and magnitude and phase threshold of impedance values in varying tissue types must be established for robust tissue analysis [194–196]. Furthermore, the complex architecture of VCA complicates analysis since the flow of electric current and therefore impedance depends on the orientation of cells [197]. Additionally, impedance measurements are affected by the anisotropic properties of nerve fibers, muscle, and blood vessels [198]. Judicious electrode configurations that mitigate anisotropic effects should be considered when applying impedance to tissue analysis [199]. The prospect of multimodal data fusion (MMDF) of temporal bioimpedance data, reflective of edema, with metabolic and cytokine profile data, and histology data to achieve VCA stratification has recently been advanced [100].

## Opportunities and future directions

While much work is still necessary for the clinical acceptance of methods other than static cold storage, evidence for effective and novel VCA preservation solutions shows promise. Introducing nutrient flow and metabolite removal may overcome consequences of ischemia. Maintaining normothermia limits tissue damage associated with cold storage. Long-term VCA outcomes stand to be improved by developments in perfusion technology including electrical myostimulation (EMS) to maintain muscle tone and reduce atrophy [204, 205] support endothelial angiogenesis [206], and support nerve regeneration [207–209]. Before these methods can be established, it is necessary to verify improvements in tissue status to evaluate the merits of novel preservation solutions. Decline in tissue viability occurs progressively during storage and is associated with molecular and physical biomarkers. Current preservation strategies strive to prolong allograft viability and mitigate rejection by reducing pathophysiologic symptoms. Ex vivo tissue analysis can provide an empirical basis for clinical decisions to discard, treat, or transplant allografts. Ultimately, robust diagnostic monitoring can complement therapeutics to expand VCA feasibility.

### Technological advancement

Given the relatively recent development of VCA surgery, progress in the diversity of tissue assessment modalities has been relatively slow, but promising. Liquid biopsies allow quantitative observation of cellular and molecular tissue changes. Noninvasive tools from solid organ transplantation have been adapted to visualize tissues of VCA on a microvascular scale. While insightful, further technological advancement is necessary for use in ex vivo tissue monitoring. Rather than using sample tissue or perfusate for proteomic and metabolomic analysis, in-line sensors complement machine perfusion to enable real-time and continuous monitoring to enable automated adaptation using perfusate supplements [70]. To meet the needs of VCA, commercial in-line sensors must be calibrated to specific models of ex vivo grafts and their perfusion conditions; moreover further improvement in their limit of detection is required to permit detection on a sub-millimolar scale. The non-invasive tools that were discussed in this review rely principally on conventional medical imaging technologies. A significant area of development for ex vivo monitoring is hardware size, as many high resolution methods lack portability. While recent technological advancements have enabled miniaturization, image analysis remains semi-quantitative and requires substantial expertise for data analysis and interpretation. A promising avenue of development leverages bioimpedance for non-invasive and continuous tissue evaluation. However, further investigation is warranted as the electrical properties of tissues are impacted by tissue composition, fluid retention, differences in preservation conditions, and other experimental variables [210].

### Graft pre-conditioning

As a step beyond VCA preservation, recent developments in machine perfusion apply graft conditioning prior to transplantation. Both immunomodulatory molecules and hematopoietic cells have been used to lower the probability of transplant rejection [18, 57, 211–216]. As discussed, VCA is highly immunogenic and presents a high risk of acute rejection. While rejection can be treated following transplantation with standard immunosuppression regimens, medication is also a significant source of non-compliance due to the high volume needed [217]. Notable instances of acute rejection have occurred in which grafts had to be removed due to non-compliance with medication [218–220]. Furthermore, long-term immunosuppression increases the risk of developing infectious complications. Immunomodulation of the VCA to immunologically condition the graft prior to transplantation may enhance graft acceptance by the recipient immune system and potentially reduce immunosuppressive treatment regimens.

## Conclusion

Vascularized composite allotransplantation is a complex and multifaceted procedure that may significantly improve patient quality of life. However, logistical challenges and heightened risks of immune rejection limit the number of feasible procedures. These challenges are compounded by damage-susceptible muscle and nerve tissues, which must remain intact to restore functional motor capabilities. The development of effective preservation methods would allow the potential to perform VCA transplantation outside the restrictive 4- to 6-h time window of current standard methods, thus expanding the pool of acceptable allografts and buying time for complex transplantation procedures without compromising graft functionality. As further research seeks to develop methods to minimize graft damage while sustaining viability for extended periods of time, advancements in status monitoring technology must establish their efficacy. Quantitative, substantive evidence for the efficacy of ex vivo perfusion and varying storage conditions are currently limited. To bridge the gap between modern technology and VCA transplantation, it is necessary to develop analytical tools that evaluate surrogate biomarkers of tissue status and connect their significance to transplant outcomes.
